# An interpretable online prediction method for remaining useful life of lithium-ion batteries

**DOI:** 10.1038/s41598-024-63160-2

**Published:** 2024-05-31

**Authors:** Zuxin Li, Shengyu Shen, Yifu Ye, Zhiduan Cai, Aigang Zhen

**Affiliations:** 1 School of Intelligent Manufacturing, Huzhou College, Huzhou, 313000 China; 2 School of Engineering, Huzhou University, Huzhou, 313000 China; 3 Zhejiang Tianneng New Materials Co., Ltd, Huzhou, 313103 China; 4 Huzhou Branch, China Telecom, Huzhou, 313000 China

**Keywords:** Lithium-ion battery, Remaining useful life, Capacity regeneration, Hybrid methods, Shapley additive explanation, Batteries, Electrical and electronic engineering

## Abstract

Accurate prediction of the remaining useful life (RUL) of lithium-ion batteries is advantageous for maintaining the stability of electrical systems. In this paper, an interpretable online method which can reflect capacity regeneration is proposed to accurately estimate the RUL. Firstly, four health indicators (HIs) are extracted from the charging and discharging process for online prediction. Then, the HIs model is trained using support vector regression to obtain future features. And the capacity model of Gaussian process regression (GPR) is trained and analyzed by Shapley additive explanation (SHAP). Meanwhile, the state space for capacity prediction is constructed with the addition of Gaussian non-white noise to simulate the capacity regeneration. And the modified predicted HIs and noise are obtained by unscented Kalman filter. Finally, according to SHAP explainer, the predicted HIs acting as the baseline and the modified HIs containing information on capacity regeneration are chosen to predict RUL. In addition, the bounds of confidence intervals (CIs) are calculated separately to reflect the regenerated capacity. The experimental results demonstrate that the proposed online method can achieve high accuracy and effectively capture the capacity regeneration. The absolute error of failure RUL is below 5 and the minimum confidence interval is only 2.

## Introduction

Widely used in electronic devices, aerospace and other fields, lithium-ion batteries play an important role in energy storage systems^1^. Over long-term usage, the performance of batteries will gradually degrade. When the state of health and lifetime of batteries reach the threshold, batteries are prone to leakage and short circuit^2^. Accurate remaining useful life (RUL) prediction provides timely information on the degree of battery aging and helps in the management of batteries, which can prevent a series of catastrophic accidents^3^. Therefore, it is critical to obtain RUL in real-time.

The RUL of lithium-ion batteries is defined as the number of charging and discharging cycles before the battery operates under certain conditions and reaches the end of life (EOL). The lifetime of batteries is terminated when the actual capacity is below 70%-80% of the rated capacity^4^. In recent years, RUL prediction methods have received great interest. Generally, the prediction methods can be classified into three categories: model-based approaches, data-driven approaches and hybrid approaches^5^. And traditional prediction methods have complete observations corresponding to the time of the prediction step, and thus these are not fully online.

Model-based approaches, such as the enhanced single particle model, can deeply analyze the electrochemical mechanism of battery operations, but the models are complicated to construct in real-time systems^6,7^. Data-driven approaches utilize the acquired observations for RUL prediction, which are known to have better generalizability. Based on the capacity degradation data of batteries, data-driven approaches, such as widely used support vector regression (SVR) and Gaussian process regression (GPR), can accurately predict RUL. These capacity-based methods can obtain accurate RUL, but are almost impossible to achieve online prediction due to the lack of battery capacity in real-time. Therefore, some researchers have extracted health indicators (HIs) from the signals that can be measured to characterize the battery aging^8,9^. Generally, the predicted results are relatively accurate based on HIs, but when the extraction of HIs is biased or even of poor quality, the prediction accuracy of RUL will be greatly affected. This online method of step prediction requires high-quality hardware for real-time acquisition of HIs. Just like SVR and GPR, the step prediction method is capable of predicting single-point results. However, it lacks the ability to accurately estimate the battery capacity in advance, thus preventing effective battery management^10,11^. Moreover, the prediction accuracy of these methods is seriously affected by capacity regeneration and noise fluctuations^12–14^.

In recent years, there have been a variety of hybrid methods, with two major categories^15^. The first type of hybrid methods is a combination of model-based and data-driven methods, which usually consists of filtering methods with equivalent circuit models and empirical degradation models for RUL prediction^16^. The battery model serves as the state equation, the output of the data-driven method is used as the observation, and the parameters of the model can be updated by the filtering to ensure the prediction accuracy^17,18^. In general, the initialization of model parameters is set empirically, which can significantly impact the convergence performance of the algorithm. The second type of hybrid methods is a combination of multiple data-driven approaches, which achieve RUL prediction by integrating different algorithms^19,20^. Wavelet transform, empirical modal decomposition and other techniques are commonly used in these hybrid methods to achieve data deconstruction with greater complexity of the algorithms^21,22^. These methods prioritize enhancing the prediction accuracy of RUL for offline conditions or online step prediction. However, they may not be able to respond promptly when the battery reaches EOL prematurely. For the online prediction, the capacity can be estimated after the HIs are predicted by hybrid methods. But the predicted HIs can hardly reflect the complete information, especially the capacity regeneration that can cause sudden fluctuations in capacity, which makes online estimation difficult^23^.

In addition, there is a concern about the interpretable analysis of data-driven methods, which can provide insights into black-box approaches. The interpretable analysis typically enables greater transparency in the decision-making process of the model. It can make the results of machine learning models more reliable and make the mechanism of the model more compatible with prior knowledge^24^. Generally speaking, the interpretation methods are classified into pre-model, in-model and post-model approaches^25^. Among them, post-model approaches, such as the Shapley additive explanation (SHAP), are able to evaluate the contribution of all features^26^. Therefore, SHAP analysis is usually used to quantify feature contributions to the model and to deepen the model interpretation^27^. As an interpretation method, SHAP analysis cannot reflect the causality, and it is also challenging to enhance the data-driven method based on the analysis.

Given the aforementioned challenges, an interpretable online prediction method is proposed. Firstly, the proposed method extracts HIs from batteries’ current, voltage and temperature signals to fully describe the battery aging. Secondly, HIs are quantified in terms of importance according to the grey relation analysis (GRA) method. The determined HIs are trained through SVR for the HIs model^28^. The predicted HIs by SVR serve as online inputs for both the capacity model of GPR and the state space of capacity prediction, respectively. On the one hand, capacity prediction based on the trained GPR model is followed by SHAP analysis. The HIs are ranked and divided into two groups by the contribution, with the criterion being more than 50% of total contributions. On the other hand, the state quantities of the state space are defined as the HIs with added Gaussian non-white noise that simulates the capacity regeneration which may occur suddenly at any time, and the observation quantity is the predicted capacity in that case. An unscented Kalman filter (UKF) is used to modify the HIs and the Gaussian non-white noise at each predicted time. Thirdly, the original predicted HIs are combined with the modified HIs based on the indexes obtained from the SHAP analysis. The original predicted HIs with greater contribution are selected as features ensuring the baseline of the predicted capacity of GPR, while modified predicted HIs are selected to provide the capacity regeneration information on the criterion of lesser contribution. Finally, the capacity can be predicted from the combined HIs and the GPR model, and the online prediction of RUL is achieved. It is noted that in the proposed method, the bounds of the prediction confidence intervals (CIs) are obtained in a different way. The lower bound can be obtained directly by calculating the 95% confidence interval from the final predicted capacity, while the upper bound needs to be calculated the interval after adding the modified Gaussian non-white noise to the final predicted capacity in order to characterize the capacity regeneration.

The main contributions of the paper are as follows:

1. Four HIs are constructed for online prediction to comprehensively characterize the battery aging, and the battery capacity is initially estimated online based on SVR and GPR.
2. A state space for capacity prediction is constructed with the addition of a Gaussian non-white noise for the capacity regeneration, and the predicted HIs and the artificial noise are modified by UKF.
3. SHAP analysis is developed to select appropriate HIs, ensuring the accuracy of capacity prediction while characterizing the capacity regeneration.
4. The upper and lower bounds of CI are developed to be obtained in two ways, while the lower bound ensures the reliability of the RUL prediction, the upper bound can show the capacity regeneration.

The rest of this paper is organized as follows: Section II proposes the interpretable hybrid method for online prediction of RUL. Section III introduces the extracted HIs and the corresponding quantitative analysis. Section IV presents the experimental results of the proposed method based on the NASA data sets. The conclusions are presented in Section V.

## Methodology

### Theoretical foundations

#### Support vector regression

SVR is a frequently utilized technique for addressing nonlinear regression problems with small data^29^. The strong generalization ability of this method is achieved by minimizing the structural risk, and the implied statistical information can be well mined when the sample size is sufficiently small^30^. The algorithm structure is shown as follows.

Training sample $D=\left\{ ({{x}_{1}},{{y}_{1}}),({{x}_{2}},{{y}_{2}}),\cdots ({{x}_{m}},{{y}_{m}}) \right\}$ is first determined, where ${{x}_{i}}\in {{R}^{d}}$ is the *d*-dimensional feature vector, ${{y}_{i}}\in R$ is the target output, and the total number of training samples is *m*. Based on the training sample, the object of SVR is to obtain a regression model that sets *f*(*x*) as close as possible to *y*. And the SVR function can be described as follows:

1 $$f(x_{i} ) = \omega ^{T} x_{i} + b$$

where $\omega$ is the weight vector and *b* is the bias value.

In SVR, the error $\varepsilon$ is defined as the difference between *f*(*x*) and *y*, and the maximum error is set to $\varepsilon _{max}$. When $\varepsilon _{max}$ is less than $\varepsilon$, $\varepsilon$ will be used to be processed instead. In other words, the prediction is accepted when the training data are centered at *f*(*x*) within the regions of width $2\varepsilon _{max}$. Therefore, the objective of SVR can be expressed as follows:

2 $$\begin{aligned} \mathop {\min }\limits _{\omega ,b}\,\frac{{1}}{{2}}{{\left\| \omega \right\| }^{{2}}} + C\mathop {\sum }\limits _{i=1}^{m}{{\ell }_{\varepsilon }}(f({{x}_{i}})-{{y}_{i}}) \end{aligned}$$

where *C* is the penalty factor, ${\ell }_{\varepsilon }$ is the insensitive loss function which is as follows.

3 $$\begin{aligned} {\ell }_{\varepsilon } (f(x),y) = \left\{ \begin{array}{*{2}{ll}} 0 &{} \left| \varepsilon \right| \le \varepsilon _{max} \\ \left| \varepsilon \right| -\varepsilon _{max} &{} \left| \varepsilon \right| >\varepsilon _{max} \\ \end{array} \right. \ \end{aligned}$$

Since the errors in the actual problem will make SVR more sparse, this paper introduces slack variables ${{\xi }_{i}}$ and $\hat{{{\xi }_{i}}}$, which reduces Eq. 2 to the following Eq. 4.

4 $$\begin{aligned} \underset{\omega ,b,{{\xi }_{i}},{{{\overset{\scriptscriptstyle \frown }{\xi }}}_{i}}}{\mathop {\min }}\,\frac{1}{2}{{\left\| \omega \right\| }^{2}}+C\mathop {\sum }\limits _{i=1}^{m}{({{\xi }_{i}}+{{{\overset{\scriptscriptstyle \frown }{\xi }}}_{i}})} \end{aligned}$$

The constraints of Eq. 4 are shown below,

5 $$\begin{aligned} s.t.\left\{ \begin{aligned}{}&{{\omega }^{T}}{{x}_{i}}+b-{{y}_{i}}\le \varepsilon _{max} +{{\xi }_{i}} \\&{{y}_{i}}-{{\omega }^{T}}{{x}_{i}}-b\le \varepsilon _{max} +{{{\overset{\scriptscriptstyle \frown }{\xi }}}_{i}} \\&{{\xi }_{i}}\ge {0}, {{{\overset{\scriptscriptstyle \frown }{\xi }}}_{i}}\ge 0, i=1,2,\cdots ,m \\ \end{aligned} \right. \end{aligned}$$

Then, Lagrange multipliers ${{\mu }_{i}}\ge 0,{{\overset{\scriptscriptstyle \frown }{\mu }}_{i}}\ge 0,{{\alpha }_{i}}\ge 0,{{\overset{\scriptscriptstyle \frown }{\alpha }}_{i}}\ge 0$ are added to give the Lagrangian function shown in Eq. 6:

6 $$\begin{aligned} \begin{aligned} L(\omega ,b,{{\xi }_{i}},{{{\overset{\scriptscriptstyle \frown }{\xi }}}_{i}},\alpha ,{{{\overset{\scriptscriptstyle \frown }{\alpha }}}_{i}},\mu ,\overset{\scriptscriptstyle \frown }{\mu })&=\frac{1}{2}{{\left\| \omega \right\| }^{2}}+C\mathop {\sum }\limits _{i=1}^{m}{({{\xi }_{i}}+{{{\overset{\scriptscriptstyle \frown }{\xi }}}_{i}})-\mathop {\sum }\limits _{i=1}^{m}{{{\mu }_{i}}{{\xi }_{i}}}}-\mathop {\sum }\limits _{i=1}^{m}{{{{\overset{\scriptscriptstyle \frown }{\mu }}}_{i}}{{{\overset{\scriptscriptstyle \frown }{\xi }}}_{i}}} \\&\quad +\mathop {\sum }\limits _{i=1}^{m}{{{\alpha }_{i}}(f({{x}_{i}})-{{y}_{i}}-\varepsilon _{max} -{{\xi }_{i}})} +\mathop {\sum }\limits _{i=1}^{m}{{{{\overset{\scriptscriptstyle \frown }{\alpha }}}_{i}}({{y}_{i}}-f({{x}_{i}})-\varepsilon _{max} -{{{\overset{\scriptscriptstyle \frown }{\xi }}}_{i}})} \\ \end{aligned} \end{aligned}$$

After taking the partial derivatives of $\omega ,b,{{\xi }_{i}},{{\overset{\scriptscriptstyle \frown }{\xi }}_{i}}$ and setting the partial derivatives to zero for Eq. 6 respectively, the new objective of SVR could be obtained as shown in Eq. 7.

7 $$\begin{aligned} \begin{aligned}{}&{\begin{aligned} \underset{\alpha ,\overset{\scriptscriptstyle \frown }{\alpha }}{\mathop {\max }}\,\mathop {\sum }\limits _{i=1}^{m}&{{{y}_{i}}({{{\overset{\scriptscriptstyle \frown }{\alpha }}}_{i}}-{{\alpha }_{i}})-\varepsilon _{max} ({{{\overset{\scriptscriptstyle \frown }{\alpha }}}_{i}}+{{\alpha }_{i}})}-\frac{1}{2}\mathop {\sum }\limits _{i=1}^{m}{\mathop {\sum }\limits _{j=1}^{m}{({{{\overset{\scriptscriptstyle \frown }{\alpha }}}_{i}}-{{\alpha }_{i}})({{{\overset{\scriptscriptstyle \frown }{\alpha }}}_{j}}-{{\alpha }_{j}})x_{i}^{T}{{x}_{j}}}} \\\end{aligned}}\\&s.t. \mathop {\sum }\limits _{i=1}^{n}{({{{\overset{\scriptscriptstyle \frown }{\alpha }}}_{i}}-{{\alpha }_{i}})} = 0, 0\le {{\alpha }_{i}}, {{\overset{\scriptscriptstyle \frown }{\alpha }}_{i}}\le C\\\end{aligned} \end{aligned}$$

From the constraints, the SVR function could be obtained as follows:

8 $$\begin{aligned} f(x)=\mathop {\sum }\limits _{i=1}^{m}{({{{\overset{\scriptscriptstyle \frown }{\alpha }}}_{i}}-{{\alpha }_{i}})x_{i}^{T}x}+b \end{aligned}$$

Considering the nonlinear feature mapping, the function could be shown as follows:

9 $$\begin{aligned} f(x)=\mathop {\sum }\limits _{i=1}^{m}{({{{\overset{\scriptscriptstyle \frown }{\alpha }}}_{i}}-{{\alpha }_{i}})\kappa ({{x}_{i}},{{x}_{j}})}+b \end{aligned}$$

where $\kappa$ is the kernel function. In this paper, common radial basis kernel function is chosen for SVR. The kernel function is listed as follows.

10 $$\begin{aligned} \kappa ({{x}_{i}},{{x}_{j}})=\exp \left( -\frac{{{\left\| {{x}_{i}}-{{x}_{j}} \right\| }^{2}}}{2{{\sigma }^{2}}}\right) ,\sigma >0 \end{aligned}$$

where $\sigma$ is the kernel width.

#### Gaussian process regression

Based on the Bayesian theory, GPR is a flexible nonparametric model^31^. The majority of systems can be modeled by using a suitable combination of Gaussian processes (GP), and the performance degradation or model mismatch could occur in the long-term predictions. The GPR model performs better in multi-step prediction, and it is applicable to modeling the aging process of batteries with strong nonlinearity^32^. The structure of GPR is shown as follows.

In the Gaussian process modeling, *f*(*x*) is considered as a set of random variables, and the mathematical representation is shown as follows:

11 $$\begin{aligned} \left\{ \begin{aligned}{}&f(x)\sim GP(m(x),{{k}_{f}}(x,{x}'))\\&m(x)=E\left[ \left( f(x) \right) \right] \\&{{k}_{f}}(x,{x}')=E\left[ \left( f(x)-m(x) \right) {{\left( f({x}')-m({x}') \right) }^{T}} \right] \\ \end{aligned} \right. \end{aligned}$$

The conventional GPR mean function is set to zero, while in this paper, the linear mean function is chosen as the mean function to obtain better long-term prediction performance. The linear mean function is shown as follows:

12 $$\begin{aligned} m(x)=a*x+b \end{aligned}$$

The covariance function must be chosen to satisfy the semi-positive definite matrix characteristic, whose main function is to quantify the relationship between points. If the input points are close to each other, the outputs should also be close. Therefore, the squared exponent is chosen as the covariance function in this paper to ensure the prediction performance. the covariance function is shown as follows:

13 $$\begin{aligned} {{k}_{f}}(x,{x}')=\sigma _{f}^{2}\exp \left( -\frac{{{(x-{x}')}^{2}}}{2{{l}^{2}}} \right) \end{aligned}$$

where $\sigma _{f}^{2}$ denotes the signal variance, and *l* denotes the characteristic length scale of each input vector.

The set of hyperparameters of the constructed model is $\theta = \left[ a,b,l,\sigma _{f}^{2} \right]$. To achieve the optimization of hyperparameters, the maximum likelihood estimation is used in this paper. The method constructs the maximum likelihood function based on the unknown parameters and the sample data. The method could obtain the optimal hyperparameters by minimizing the negative log-likelihood function, which is shown as Eq. 14.

14 $$\begin{aligned} {{\theta }_{opt}}=\underset{\theta }{\mathop {\arg \min \left( \text {NLML}\right) }} \end{aligned}$$

where NLML is shown as follows.

15 $$\begin{aligned} \begin{aligned} \text {NLML}&=-\log p(\left. y \right| x,\theta )\\&=\frac{1}{2}{{y}^{T}}{{\left[ {{K}_{f}}(x,x)+\sigma _{n}^{2}{{I}_{n}} \right] }^{-1}}y+\frac{1}{2}\log \left( \det ({{K}_{f}}(x,x)+\sigma _{n}^{2}{{I}_{n}}) \right) +\frac{n}{2}\log 2\pi \\ \end{aligned} \end{aligned}$$

To initialize the parameters, the conjugate gradient algorithm^33^ is used to solve the equations, and the maximum value of the objective function can be obtained by taking partial derivatives of Eq. 15. The expressions are shown as Eq. 16.

16 $$\begin{aligned} \left\{ \begin{aligned} \frac{\partial }{\partial {{\theta }_{k}}}&\log p(\left. y \right| x,\theta )=\frac{1}{2}\!tr\!\left\{ \left[ \!\alpha {{\alpha }^{T}}\!\!-\!{{\left( {{K}_{f}}(x,x)\!+\!\sigma _{n}^{2}{{I}_{n}} \right) }^{\!-1\!}}\frac{\partial ({{K}_{f}}(x,x)\!+\!\sigma _{n}^{2}{{I}_{n}})}{\partial {{\theta }_{k}}}\! \right] \right\} \\ \alpha =&{{({{K}_{f}}(x,x)+\sigma _{n}^{2}{{I}_{n}})}^{-1}}y \\ \end{aligned} \right. \end{aligned}$$

where *tr* denotes the trace of the matrix, and ${{\theta }_{k}}$ is an element of the set ${{\theta }}$ of hyperparameter.

The observation equation is defined as Eq. 17, where *v* is the Gaussian white noise, $v \sim N(0,\sigma _{n}^{2})$. Thus the prior distribution of *y* could be shown as Eq. 18.

17 $$\begin{aligned} y= & {} f(x)+v \end{aligned}$$

18 $$\begin{aligned} y\sim & {} N\left( m(x),{{K}_{f}}(x,x)+\sigma _{n}^{2}{{\delta }_{ij}} \right) \end{aligned}$$

In Eq. 18, ${{\delta }_{ij}}$ is the Dirac function, ${{\delta }_{ij}}={{I}_{n}}$. Given the test input ${{x}^{*}}$, the joint prior distribution of *y* and the prediction set ${{y}^{*}}$ are shown as follows:

19 $$\begin{aligned} \left[ \begin{aligned}{}&y\!\\&{{y}^{*}}\! \\ \end{aligned} \right] \!\sim \! N\!\left( \left[ \! \begin{aligned}{}&m(x) \!\\&m({{x}^{*}}) \\ \end{aligned} \right] ,\left[ \begin{matrix} {{K}_{f}}(x,x)\!+\!{{I}_{n}}\sigma _{n}^{2} &{} {{K}_{f}}(x,{{x}^{*}}) \\ {{K}_{f}}{{(x,{{x}^{*}})}^{T}} &{} {{K}_{f}}({{x}^{*}},{{x}^{*}}) \\ \end{matrix} \right] \right) \end{aligned}$$

where ${{K}_{f}}(x,x)$ is the covariance matrix of the training data, and ${{K}_{f}}(x,{{x}^{*}})$ is the covariance matrix of the training input and the test input, ${{K}_{f}}(x,{{x}^{*}})={{K}_{f}}{{({{x}^{*}},x)}^{T}}$, and ${{K}_{f}}({{x}^{*}},{{x}^{*}})$ is the covariance matrix of the test data. The analytic form of the derived posterior distribution is shown as Eq. 20

20 $$\begin{aligned} p(\left. {{y}^{*}} \right| x,y,{{x}^{*}})=N\left( \left. {{y}^{*}} \right| {{{{\hat{y}}}}^{*}},{\text {cov}}({{y}^{*}}) \right) \end{aligned}$$

The predicted average and the predicted covariance are shown as follows:

21 $$\begin{aligned} {{{\hat{y}}}^{*}}= & {} {{K}_{f}}{{(x,{{x}^{*}})}^{T}}\left[ {{\left[ {{K}_{f}}(x,x)+\sigma _{n}^{2}{{I}_{n}} \right] }^{-1}}(y-m(x)) \right] +m({{x}^{*}}) \end{aligned}$$

22 $$\begin{aligned} {\text {cov}}({{y}^{*}})= & {} \!-\!{{K}_{f}}{{(x,{{x}^{*}})}^{T}}{{\left[ {{K}_{f}}(x,x)\!+\!\sigma _{n}^{2}{{I}_{n}} \right] }^{-1}}{{K}_{f}}(x,{{x}^{*}})+{{K}_{f}}({{x}^{*}},{{x}^{*}}) \end{aligned}$$

The predicted average ${{{\hat{y}}}^{*}}$ is taken as the output of the prediction model, while ${\text {cov}}({{y}^{*}})$ reflects the uncertainty of the prediction model. According to Eq. 23, the 95% confidence intervals *CI* could be calculated.

23 $$\begin{aligned} CI={{{\hat{y}}}^{*}}\pm 1.96\cdot {\text {cov}}({{y}^{*}}) \end{aligned}$$

#### SHAP analysis

SHAP analysis was first introduced into the field of machine learning as a method in cooperative game theory in^34^. The method is able to quantify the contribution of each feature to the trained machine learning model *f*(*x*), which is an interpretation method independent of the type of model. The SHAP value $\phi _{i}$ of each feature $x_{i}$ is calculated as shown in Eq. 24.

24 $$\begin{aligned} {{\phi }_{i}}=\!\!\!\mathop {\sum }\limits _{S\subseteq F\backslash \{i\}}\!\!{\frac{\left| S \right| !\left( \left| F \right| -\left| S \right| -1 \right) !}{\left| F \right| !}}\left[ {{f}_{S\cup \{i\}}}\left( {{x}_{S\cup \{i\}}} \right) -{{f}_{S}}\left( {{x}_{S}} \right) \right] \end{aligned}$$

where *F* is the set of all features, $S\subseteq F\backslash \{i\}$. For each feature $x_{i}$, the SHAP analysis involves selecting each set containing $x_{i}$ to calculate its marginal contribution. And the SHAP value for $x_{i}$ is the weighted average of all possible differences.

#### Unscented Kalman filter

Kalman filter is a recursive algorithm for estimating the current state using the prior state and the current measured signal. To solve the nonlinearity, UKF achieves Gaussian density approximation by obtaining Sigma points through the unscented transformation^35^. The pseudo code of UKF algorithm is presented in Algorithm 1.

In Algorithm 1, *f* is the state equation, *h* is the observation equation, *W* and *V* are mutually independent Gaussian white noises with covariance matrices *Q* and *R*, respectively. *n* is the dimension of the state, $j=1, 2, \cdots , 2n+1$. $\lambda$ is the parameter of scaling, and *weight* is the weight corresponding to the $2n+1$ Sigma points.

**Algorithm 1:**
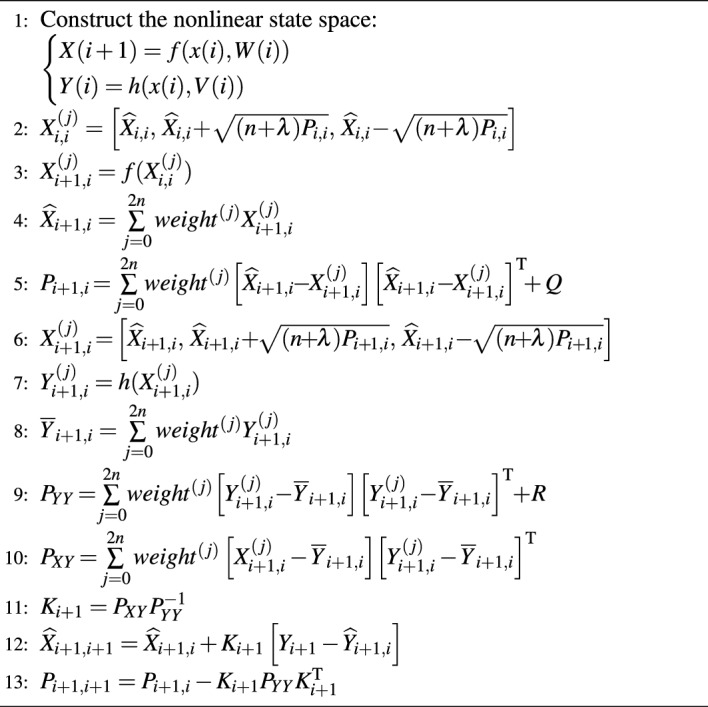
The unscented Kalman filter algorithm

### Interpretable online prediction method

**Figure 1:**
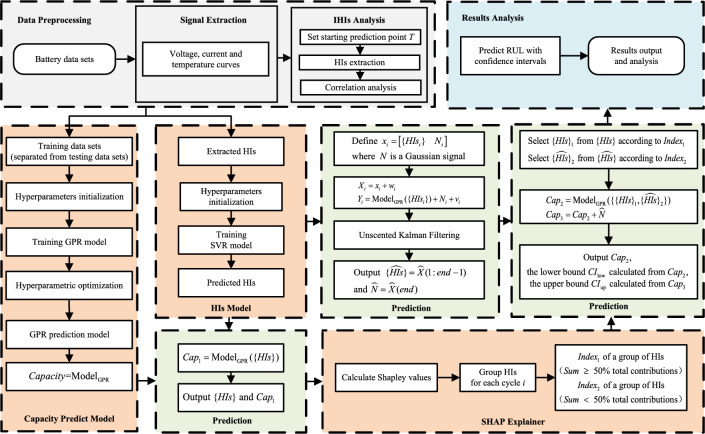
The flowchart of proposed method.

In this section, an interpretable method for online RUL prediction is proposed, and the flowchart of the proposed method is shown in Fig. 1. The process is as follows:

1. ***Feature extraction and analysis:*** From constant-current (CC) charging signals, constant-voltage (CV) charging signals, CC discharge signals, and temperature signals, appropriate HIs are extracted to characterize the aging of lithium-ion batteries. The GRA method is used to quantitatively assess the reasonableness and validity of the extracted HIs.
2. ***Data processing:*** Normalization of sample data.
3. ***Parameters initialization:*** The starting prediction point *T* and the battery capacity failure threshold $Cap_{EOL}$ are set.
4. ***HIs modeling:*** Based on SVR, HIs model is constructed and the predicted HIs can be obtained and as the future input in online prediction. The detailed steps are as follows:
  - According to *T*, the data are divided into the training set and the test set.
  - The penalty coefficient *C* and kernel width $\sigma$ of SVR are initialized.
  - The HIs model is trained using the training set $\left\{ i,HI{{s}_{tr,i}} \right\} _{i}^{T-1}$, where *i* denotes the *i*-th charging and discharging cycle in the training data sets, $HI{{s}_{tr,i}}$ denotes the HIs corresponding to the *i*-th cycle.
  - As the predicted HIs after the *T* point, $\left\{ HIs \right\}$ could be obtained by the trained HIs model.
5. ***Capacity prediction modeling:*** Taking the historical HIs as the input to the capacity model and the battery capacity as the output, the relationship between HIs and the battery capacity is modeled based on GPR. The detailed steps are as follows:
  - According to *T*, the data are divided into the training set and the test set.
  - The hyperparameters $\theta = \!\![\!\!\text { }a,b,{{\sigma }_{f}},l]$ of GPR are initialized.
  - The capacity model is trained with the training set $\left\{ HI{{s}_{tr,i}},Ca{{p}_{tr,i}} \right\} _{i}^{T-1}$, where $Ca{{p}_{tr,i}}$ denotes the *i*-th battery capacity value. The model parameters are optimally selected by taking the conjugate gradient algorithm.
  - As the capactiy prediction model, $\text {Model}_{\text {GPR}}$ can be obtained.
6. ***Unscented Kalman filtering:*** For the actual battery capacity in the training data sets, which consists of the capacity of degradation and regeneration and the measurement noise. Obtained by Gaussian smoothing and polynomial fitting of the observation, the fitted curve of capacity can reflect the capacity degradation, while the fitting error characterizes the effect of capacity regeneration on battery aging. Therefore, a Gaussian non-white noise *N* is introduced to characterize the effect of capacity regeneration with the average and variance in terms of the fitting error. And *N* is added as the state quantity to the state space, then the state space can be constructed as Eq. 25.
  25 $$\begin{aligned} \left\{ \begin{aligned}{}&X_{i}=\left[ \left\{ HIs_{i}\right\} , N_{i}\right] +w_{i} \\&Y_{i}=\text {Model}_{\text {GPR}}(\left\{ HIs_{i}\right\} )+N_{i}+v_{i} \\ \end{aligned} \right. \end{aligned}$$
  where $w{\sim }{N(0,Q)}$ is the state process noise and $v{\sim }{N(0,R)}$ is the corresponding observation noise.   Using UKF, the added noise *N* and the original predicted HIs $\left\{ HIs \right\}$ are modified together depending on the capacity of $\text {Model}_{\text {GPR}}$. The modified HIs $\{ {\widehat{HIs}} \}$ and the modified noise ${\hat{N}}$ are obtained. This capacity prediction model depends on the $\text {Model}_{\text {GPR}}$, while the capacity regeneration simulated with Gaussian non-white noise *N* occurs at a higher frequency and its characterization of the effect of capacity degradation is incomplete. However, the advantage is that the capacity regeneration simulated by *N* is sudden and superimposable, which is consistent with the actual phenomenon and can provide guidance. Moreover, by the closed-loop of UKF, the modified HIs can reflect the characteristics of capacity regeneration, and the noise *N* can also be modified in amplitude and frequency.
7. ***SHAP explainer:*** From the original predicted HIs and the trained $\text {Model}_{\text {GPR}}$, the shapley values at each cycle *i* of features are calculated according to Eq. 24. Based on the obtained SHAP values, the HIs at each cycle are ranked according to the percentage of contribution. Then, HIs are combined and divided into two groups based on the criteria of the minimum number of features and their total contributions exceeding 50% of the total contributions of all HIs. Specifically, at each cycle *i*, the HI with the largest SHAP value is selected first followed by recursive addition of features of the same class (whose SHAP values are of the same sign) until the total contributions of the selected HIs exceed 50% of the total contributions of all HIs, which is treated as group I and gets the corresponding labels *Index*1, while the remaining features are treated as group II with the labels *Index*2. The labels *Index*1 and *Index*2 are for the subsequent selection of HIs. Group I, with more contributions, is to retain the portion of the original predicted HIs to maintain the output of $\text {Model}_{\text {GPR}}$ stable and reliable, while group II, with fewer contributions, will be replaced by the modified HIs to add the information of capacity regeneration.
8. ***RUL prediction:*** After the above steps, the $\text {Model}_{\text {GPR}}$, the original predicted HIs $\left\{ HIs \right\}$, the modified HIs $\{ {\widehat{HIs}} \}$ with the noise ${\hat{N}}$, *Index*1 and *Index*2 are obtained.   For each cycle *i*, HIs are selected from $\left\{ HIs \right\}$ and $\{ {\widehat{HIs}} \}$ according to *Index*1 and *Index*2, respectively, to incorporate the information of capacity regeneration while guaranteeing the trend of predicted capacity. The final combined HIs are $\{\{ HIs \}_{1}, \{ {\widehat{HIs}} \}_{2}\}$ and are used as inputs to $\text {Model}_{\text {GPR}}$ to obtain the final predicted capacity $Cap_{2}$. Based on $Cap_{2}$, the lower bound of CIs, $CI_{\text {low}}$, can be calculated according to Eq. 23, while the calculation of the upper bound $CI_{\text {up}}$ needs to be first calculated according to Eq. 26 to obtain the predicted capacity $Cap_{3}$, and then calculated according to Eq. 23.
  26 $$\begin{aligned} Cap_{3}=Cap_{2}+{\hat{N}} \end{aligned}$$
  Since $Cap_{2}$ contains less information about capacity regeneration, $Cap_{2}$ is more reliable compared to $Cap_{3}$, and $CI_{\text {low}}$ is calculated using $Cap_{2}$ to provide early warning of battery failure. $Cap_{3}$ is interspersed with more information about artificial capacity regeneration, and it is only used to calculate $CI_{\text {up}}$ to guide the use of batteries in which capacity regeneration is likely to occur and to improve the usage efficiency. When the predicted capacity $Ca{{p}_{2}}$ is less than $Ca{{p}_{EOL}}$, the RUL prediction results and the prediction confidence intervals are output.
9. ***Analysis of experimental results.***

## Data preprocessing

### Data description

The experimental data of lithium-ion batteries were acquired from NASA Ames Prognostics Center of Excellence (PCOE)^36^. The data in this study include three 18650 lithium-ion batteries (B5, B6 and B7), which were tested in three different operating modes (CC and CV charge mode, CC discharge mode and impedance measurement mode).

During CC and CV charging, the batteries were charged at a constant current of 1.5A until the battery voltage reached 4.2V, and then continued to be charged in CV until the charging current dropped to 20mA. During CC discharging, all three batteries, B5, B6 and B7 were first discharged at a constant current of 2A until the battery voltage dropped to 2.7V, 2.5V and 2.2V, respectively. The charge and discharge process was repeated to conduct the accelerated aging tests of the battery. And after each charge-discharge cycle, the battery impedance was monitored by electrochemical impedance spectroscopy, scanned from 0.1Hz to 5kHz.

### HIs extraction

The battery aging can be directly characterized by capacity and impedance, but these two indicators cannot be easily measured online due to the complex operation with expensive cost^37^. The aging can also be reflected in the variation of the observations that can be measured online, namely as HIs^38^. Therefore, it is essential to extract appropriate HIs from the observations as input features for RUL prediction.

**Figure 2:**
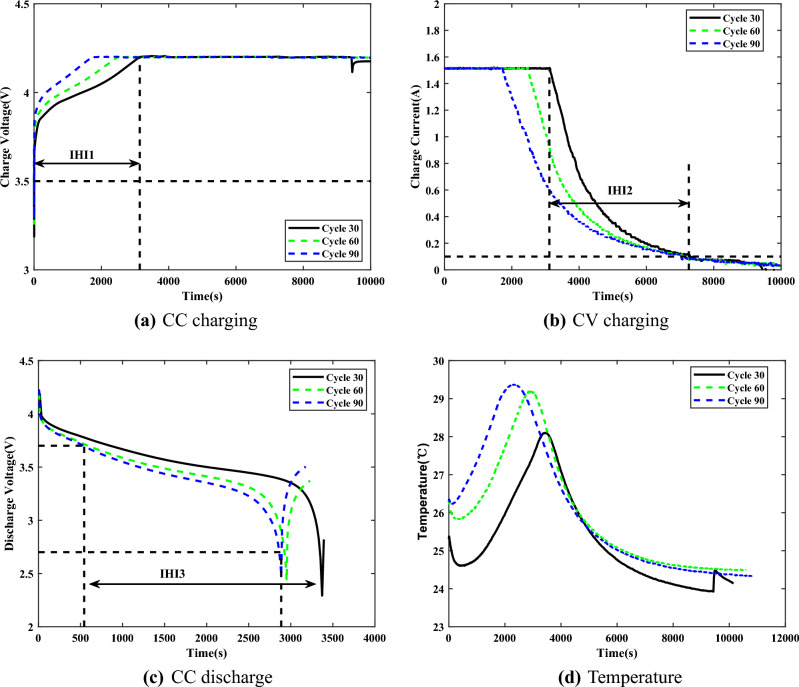
The voltage, current and temperature signals.

**Figure 3:**
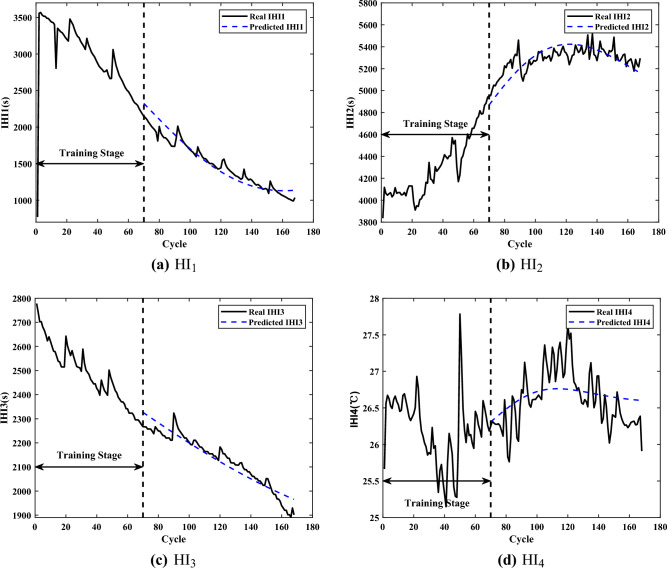
The extracted HIs and the predicted HIs.

Among the battery operations, researchers usually extract HIs from the charging or discharging process, which is defective in characterizing the battery aging^39,40^. In this paper, four appropriate HIs are extracted for online prediction, in which the effect of temperature on aging is included. The following is the analysis of the extracted HIs, taking the B6 battery as an example. Figure 2 shows the signals of B6 at different cycles. The extracted HIs and the predicted HIs with cycle numbers are shown in Fig. 3.

27 $$\begin{aligned} {{\xi }_{n}}(i)=\frac{{\mathop {\min }\limits _{n}}\,\underset{i}{\mathop {\min }}\,\left| {{x}_{0}}(i)-{{x}_{n}}(i) \right| +\alpha \underset{n}{\mathop {\max }}\,{\mathop {\max }\limits _{i}}\,\left| {{x}_{0}}(i)\!-\!{{x}_{n}}(i) \right| }{\left| {{x}_{0}}(i)-{{x}_{n}}(i) \right| +\alpha {\mathop {\max }\limits _{n}}\,{\mathop {\max }\limits _{i}}\,\left| {{x}_{0}}(i)-{{x}_{n}}(i) \right| } \end{aligned}$$

- ***HI***_1_: ***The time interval of equal charging voltage difference***^41^. In CC charging, Fig. 2a shows the variation of charging voltage for different cycles. It can be seen that the charging time gradually decreases with the number of charging times, which is due to the deepening of battery polarization . In consideration of practical applications, most users do not wait until their devices run out of energy before charging. The charging time from 3.5V to 4.2V in CC charging, is used as HI_1_ to describe the health of the battery, and the extracted series of equal charging voltage rise is shown in Fig. 3a.
- ***HI***_2_: ***The time interval of equal charging current difference***^2^. The loss of lithium-ions is more in CV charging. From Fig. 2b, the change rate of current gradually slows down as the battery gradually ages. This causes a tendency for the charging time to increase during CV charging, indicating that battery aging severely affects the lithium-ion embedding. Therefore, the time interval from the start of CV charging until the current drops to 100mA is chosen as HI_2_, shown in Fig. 3b.
- ***HI***_3_: ***The time interval of equal discharging voltage difference***^42^. With repeated charging and discharging, the battery in devices gradually degrades and the usage time of devices becomes shorter, which reflects the decay of the maximum battery capacity. Figure 2c shows the variation of the discharge voltage of battery in different cycles. The curve variation is consistent with the capacity decay trend. Therefore, the time interval of equal discharging voltage drop is extracted as HI_3_, shown in Fig. 3c, to characterize the battery aging.
- ***HI***_4_: ***The average temperature of equal charging current difference***^43^. Temperature is an important indicator of battery aging and can directly reflect the battery impedance, which is mainly composed of a combination of Joule heat and electrochemical reaction heat. Figure 2d shows the temperature variation of the battery during charging at different cycles. In CV charging, the temperature decreases due to the combination of heat dissipation, irreversible exotherm and heat absorption by the electrochemical reaction, which better reflects the battery aging. Therefore, the average temperature from the beginning of CV charging till the current reaches 100mA is extracted as HI_4_, shown in Fig. 3d.

### Grey relation analysis

GRA is developed from gray system theory, which mathematically quantifies the geometric relationship between factors with lower requirements for the quality of data^44^. The strength of the relationship between the factors can be assessed by calculating the gray correlation.

In this paper, GRA is used to quantify the correlations between the extracted HIs and the capacity. The steps of GRA are shown below:

1. ***Determination of the analysis sequence:*** The battery capacity sequence is used as the reference sequence ${{X}_{0}}=\left\{ \left. {{x}_{0}}(i) \right| i=1,2,\cdots ,k \right\}$, and the extracted HIs are used as the comparison sequence ${{X}_{n}}=\left\{ \left. {{x}_{n}}(i) \right| i=1,2,\cdots ,k \right\}$, where the sequence length is *k* and the number of comparison sequence vectors is *n*.
2. ***Data reprocessing:*** The original data are normalized.
3. ***Calculation of the correlation coefficient:*** The correlation coefficient between the points of sequence ${X}_{0}$ and ${X}_{n}$ is calculated by Eq. 27, where $\alpha$ is the resolution coefficient, $0<\alpha <1$, and $\alpha$ is set to 0.5 in this paper.
4. ***Correlation Calculation:*** The correlation between ${{X}_{0}}$ and ${{X}_{N}}$ can be calculated by Eq. 28, where ${{r}_{n}}$ represents the degree of correlation between the HIs and the capacity. The closer ${{r}_{n}}$ is to 1, the stronger the correlation between the sequences.
  28 $$\begin{aligned} {{r}_{n}}=\frac{1}{n}\mathop {\sum }\limits _{i=1}^{n}{{{\xi }_{n}}(i)} \end{aligned}$$

Table 1 shows the correlations between the four HIs and the capacity for each of the three batteries. From Table 1, it can be seen that the extracted HIs have a high correlation with the battery capacity. The correlations are all higher than 0.68, which can be used to characterize the battery aging.

**Table 1:** The correlation between the HIs and the capacity.

Numbers	${{r}_{n}}$ of HIs			
	HI_1_	HI_2_	HI_3_	HI_4_
B5	0.8037	0.7099	0.9270	0.8044
B6	0.7161	0.6869	0.8853	0.7776
B7	0.8647	0.7518	0.9395	0.8332

## Predicion results and analysis

### Evaluation criterion

To verify the interpretable online prediction method proposed in this paper, the root mean square error (RMSE), the mean absolute percentage error (MAPE), the absolute error (AE), and the relative error (RA) are used to evaluate the performance of the proposed method, and their expressions are shown as follows.

29 $$\begin{aligned} \text {RMSE}= & {} \sqrt{\frac{1}{n}\mathop {\sum }\limits _{k=1}^{n}{{{({{Q}_{k}}-{\hat{Q}}_{k}^{*})}^{2}}}} \end{aligned}$$

30 $$\begin{aligned} \text {MAPE}= & {} \frac{1}{n}\mathop {\sum }\limits _{k=1}^{n}{\left| \frac{({\hat{Q}}_{k}^{*}-{{Q}_{k}})}{{{Q}_{k}}} \right| }\times 100 \end{aligned}$$

31 $$\begin{aligned} \text {AE}= & {} \left| RU{{L}_{true}}-RU{{L}_{predicted}} \right| \end{aligned}$$

32 $$\begin{aligned} \text {RA}= & {} 1-\frac{\left| RU{{L}_{true}}-RU{{L}_{predicted}} \right| }{RU{{L}_{true}}} \end{aligned}$$

where ${{Q}_{k}}$ and ${\hat{Q}}_{k}^{*}$ denote the true value and the predicted value, respectively, and *n* is the total number of ${\hat{Q}}_{k}^{*}$. $RU{{L}_{true}}$ is the actual RUL and $RU{{L}_{predicted}}$ is the predicted RUL. The 95% confidence interval shown below is used to express the uncertainty of the prediction results.

33 $$\begin{aligned} CI={\hat{Q}}_{k}^{*}\pm 1.96\times {{\sigma }^{2}}(Q_{k}^{*}) \end{aligned}$$

RMSE is an important indicator for most predictions, which reflects the overall performance of the prediction method, while AE, RA and CIs are more significant in RUL prediction. AE and RA directly correspond to the error in predicted capacity at the time of actual battery failure. And CIs are able to alert the user well in advance of the battery failure, guiding the battery to be utilized efficiently.

### HIs prediction results

In this section, the experiments are conducted using the battery data sets (B5, B6 and B7) from NASA, and three different starting prediction points (*T*=70, *T*=80 and *T*=90) are set for each experiment. The data before *T* is used as the training set, the number of battery charging and discharging cycles is used as the training input, and the HIs are used as the training output. Based on SVR, the HIs model is constructed, and the predicted HIs after *T* can be obtained.

Taking the B6 battery as an example, Fig. 3 shows the predicted results of the HIs when the starting prediction point *T* is 70. The overall changes of the real HI and the predicted HI mostly converge, which indicates that the HI model can capture the trend of HIs well and reflect the battery aging.

The evaluation of the HIs prediction results for the three batteries is shown in Table 2. The largest RMSE in HI_1_ is found for B6 battery, and the largest RMSE in HI_2_ is found for B7 battery, both of which have values over 100. However, due to the large magnitudes of HI_1_ and HI_2_, their corresponding MAPEs are both below 0.056. And the minimum MAPE is 0.004 in HI_3_. As *T* goes backward and the number of training samples increases, the RMSE and MAPE of prediction results get smaller, which means that the prediction results become more accurate. The only exception is that the prediction of HI_3_ for the B6 battery worsens at $T=90$, which is caused by the overfitting phenomenon. The prediction error of HIs model at different starting prediction points *T* is relatively small and the prediction performance is stable. Therefore, the predicted HIs can characterize the battery aging and replace the future data of aging for the online prediction.

**Table 2:** Experimental results of predicted HIs.

Numbers	T	HI_1_		HI_2_		HI_3_		HI_4_	
		RMSE	MAPE	RMSE	MAPE	RMSE	MAPE	RMSE	MAPE
B5	70	80.64	0.029	74.88	0.014	13.46	0.005	0.318	0.009
	80	57.19	0.021	69.17	0.010	11.47	0.004	0.246	0.008
	90	37.93	0.015	55.12	0.008	9.720	0.004	0.238	0.007
B6	70	110.0	0.056	91.51	0.014	35.62	0.014	0.333	0.010
	80	77.62	0.038	85.19	0.012	32.11	0.012	0.248	0.008
	90	53.19	0.028	53.73	0.008	41.82	0.015	0.237	0.007
B7	70	82.03	0.025	134.7	0.025	13.46	0.005	0.371	0.011
	80	67.84	0.024	131.2	0.025	12.01	0.004	0.295	0.009
	90	33.72	0.012	127.8	0.024	9.863	0.004	0.269	0.008

### UKF filtering

Before constructing the state space and filtering, the actual capacity in the training data sets needs to be processed. The observation noise in the data is first removed by Gaussian smoothing, and then the polynomial fitting is utilized to obtain the degradated capacity that does not contain the information of capacity regeneration. The average and variance of the error between the fitted curve and the actual capacity are input to the state space as parameters of the Gaussian non-white noise for filtering.

**Figure 4:**
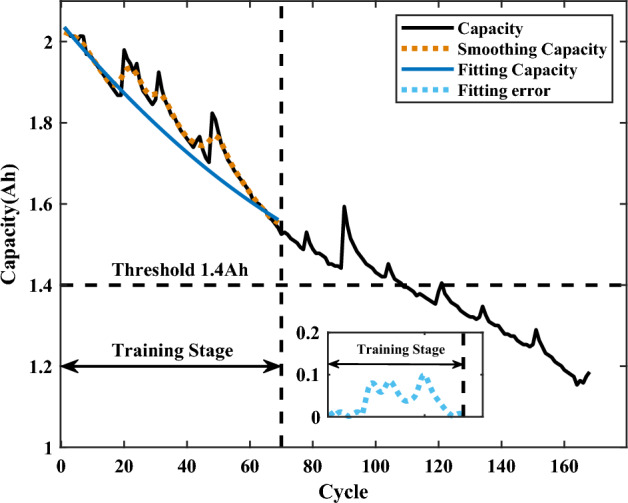
The smoothing and fitting capacity.

**Figure 5:**
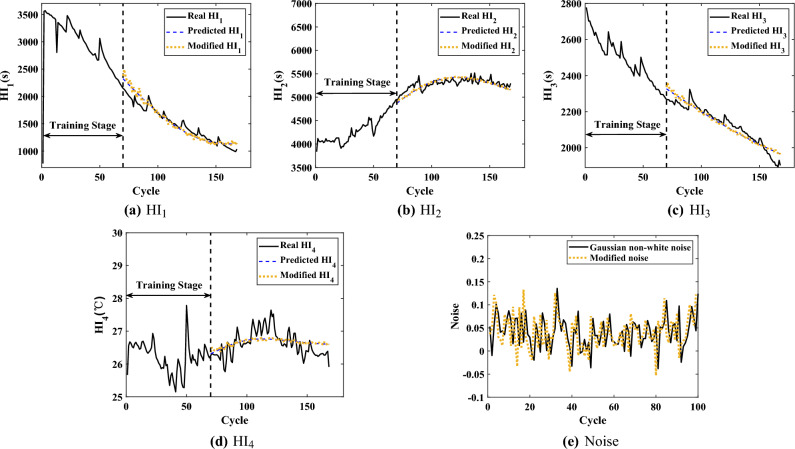
The modified HIs and the modified noise.

As an example, Fig. 4 shows the smoothing and fitting results of the actual capacity of the B6 battery. From Fig. 4, it can be seen that the smoothing capacity effectively removes the measurement noise from the original signal, while the fitted curve only retains the degradated capacity, and the corresponding fitting error can reflect the capacity regeneration. The Gaussian non-white noise is defined as $N_{i}{\sim }{N(mean_{f},Q_{f})}$, where $mean_{f}$ and $Q_{f}$ are the mean and variance matrices of the fitting error, respectively. The filtering of the UKF is then performed, and Fig. 5 shows the modified HIs and the modified Gaussian non-white noise. From Fig. 5a and d, the modified HIs are close to the real HIs in variation, and the modified HIs have obtained the information of capacity regeneration on the basis of the original predicted HIs

### SHAP explaination

**Figure 6:**
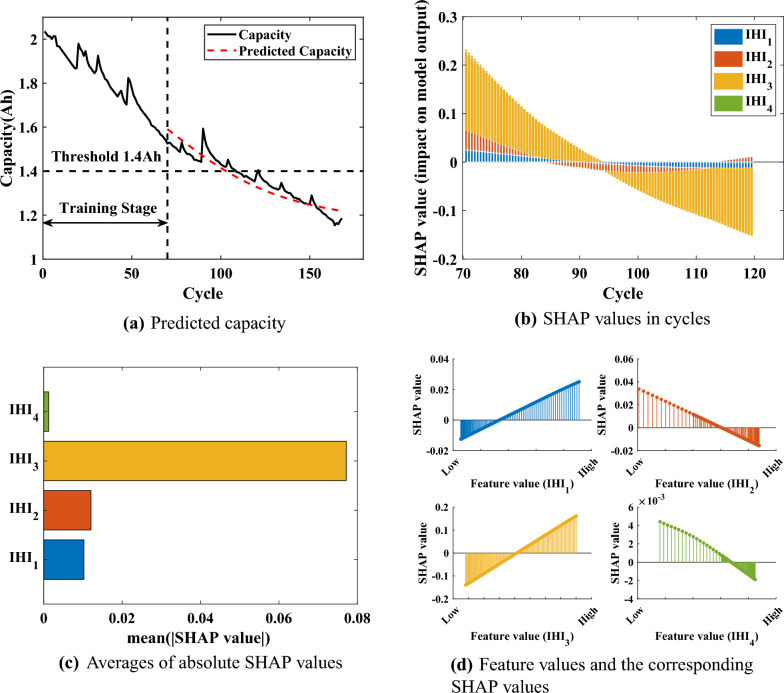
Feature importance plot.

From the original predicted HIs, the capacity model of GPR is trained, and the SHAP analysis is performed on the trained model. Again using the B6 battery as an example, Fig. 6 shows the predicted capacity of $\text {Model}_{\text {GPR}}$ with the results of the SHAP analysis. For SHAP values, their positive or negative sign does not correspond to a good or bad causality of features, but only characterizes the ability of features to contribution. Thus, the absolute SHAP values can visualize the influence of HIs on $\text {Model}_{\text {GPR}}$.

As shown in Fig. 6b and c, the SHAP values of HIs keep changing with the battery aging. The SHAP values of the HIs showed a significant shift when the cycle *i* was 80 to 100. Comprehensively, HI_3_ is the most influential among all HIs, while HI_4_ is the smallest. Figure 6d shows the variation of SHAP values in relation to the feature values. Each dotted line is a prediction sample, and thus the density of the lines indicates the distribution of the feature values. Among the samples, HI_1_ has more points with smaller feature values, whose corresponding SHAP values play a negative influence, while the contrary is the case for HI_2_ and HI_4_. HI_3_ has a more homogeneous distribution of feature values with the corresponding SHAP values, which plays a more important role in $\text {Model}_{\text {GPR}}$. In addition, in the gray correlation analysis, HI_2_ has the lowest correlation with the battery capacity, while in the SHAP analysis, the original predicted HI_2_ is second only to HI_3_ in terms of its overall contribution to $\text {Model}_{\text {GPR}}$. The result illustrates that the original predicted HI_2_ obtained by SVR contains more information about the battery aging.

After SHAP analysis, The labels *Index*1 and *Index*2 can be obtained to get the final HIs, $\{\{ HIs \}_{1}, \{ {\widehat{HIs}} \}_{2}\}$. Since the traditional RUL prediction is offline or the online step prediction, there are no future HIs (after *T*) as feature input in the online prediction. In this paper, the predicted HIs are obtained by SVR, which are smooth compared to the real HIs. Comparing with the prediction results on battery capacity in Fig. 6, the original predicted HIs contain the most information on battery degradation, while losing the part of the information on capacity regeneration and observation noise. The results of SHAP analysis are able to quantify the amount of information on battery degradation contained in different HIs at different future cycles, respectively. For the B6 battery, the original predicted HI3 contains the most information of battery degradation and HI4 the least during the whole prediction cycles. By retaining enough degradation information at different future cycles, a reliable degradation capacity could be predicted. Therefore, by combining the original predicted HIs with the modified HIs, $\{\{ HIs \}_{1}, \{ {\widehat{HIs}} \}_{2}\}$ contains both the majority of information on capacity degradation and capacity regeneration.

### RUL prediction results

Based on $\{\{ HIs \}_{1}, \{ {\widehat{HIs}} \}_{2}\}$, the battery capacity can be predicted by $\text {Model}_{\text {GPR}}$. When the predicted capacity is reduced to the threshold $Ca{{p}_{EOL}}$, the RUL prediction results become the output. Additionally, the CIs can serve as an early warning when their lower bound reaches $Ca{{p}_{EOL}}$, while their upper bound of the capacity regeneration can directly guide the usage of the battery. In this section, three batteries (B5, B6 and B7) are samely used for experiments to verify the validity and advantages of the proposed method. Three starting prediction points ($T=70$, $T=80$ and $T=90$) are set for each set of experiments. Compared with the traditional scaling of data, the data is divided in this way in order to verify the proposed method when capacity regeneration occurs at $T=90$. The rated capacity of the batteries is 2Ah, and $Ca{{p}_{EOL}}$ is set to 1.4Ah. It should be noted that the $Ca{{p}_{EOL}}$ of B7 battery is set to 1.5Ah because the capacity of the B7 battery did not degrade to reach 1.4Ah.

**Table 3:** Experimental results of RUL online prediction for B5 battery.

*T*	Method	$\text {RUL}_{\text {true}}$	$\text {RUL}_{\text {pre}}$	RMSE	AE	RA	Uncertainty	CI
70	M1	55	51	0.0203	4	0.9273	[45 47]	2
	M2		50	0.0200	5	0.9091	[43 60]	17
	M3		45	0.0386	10	0.8182	[34 62]	28
80	M1	45	43	0.0208	2	0.9556	[39 44]	5
	M2		44	0.0222	1	0.9778	[37 54]	17
	M3		28	0.0816	17	0.6222	[21 35]	14
90	M1	35	34	0.0193	1	0.9714	[32 34]	2
	M2		37	0.0226	2	0.9429	[30 45]	15
	M3		27	0.0316	8	0.7714	[18 38]	20

**Figure 7:**
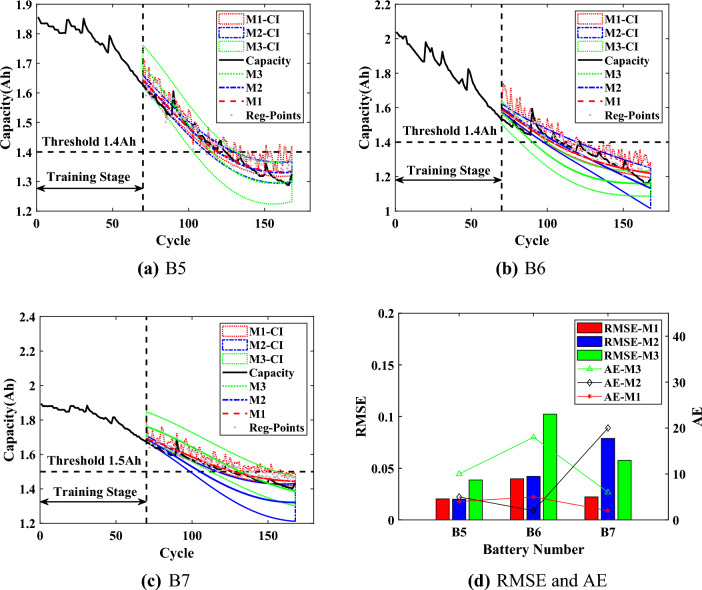
Online prediction results of RUL when $T=70$.

**Figure 8:**
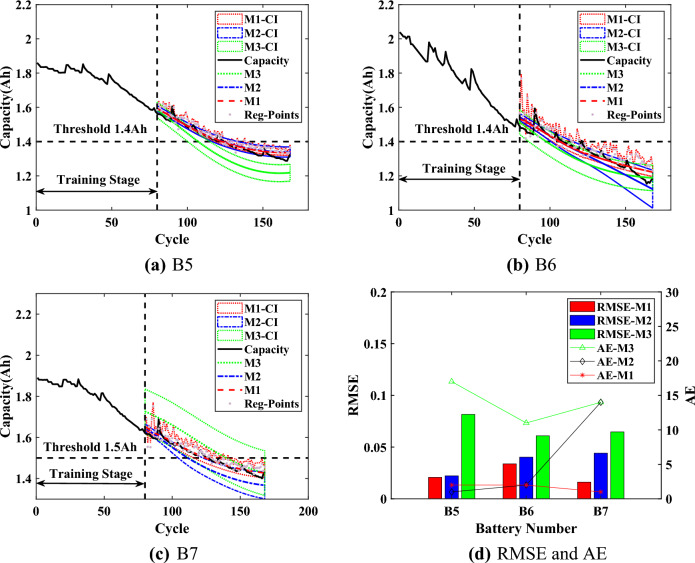
Online prediction results of RUL when $T=80$.

**Figure 9:**
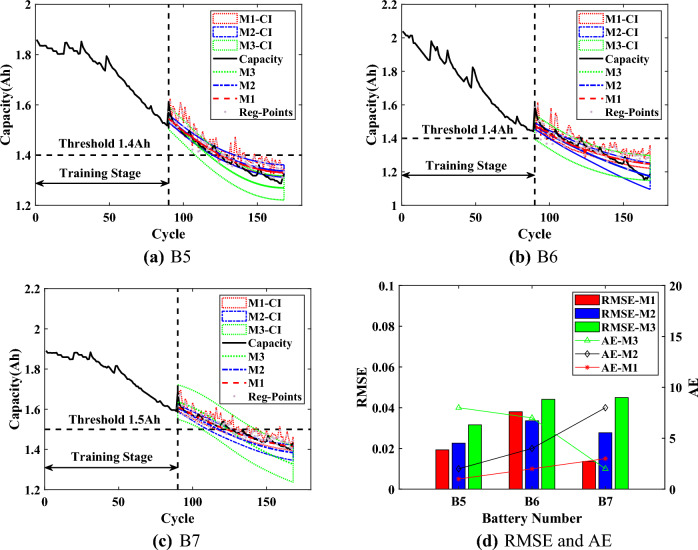
Online prediction results of RUL when $T=90$.

For comparison, the capacity prediction models all use the predicted HIs (which are obtained through SVR) as the input. The Model I (M1) is the proposed interpretable online prediction method. The Model II (M2) directly uses the predicted HIs of SVR to train the capacity model of GPR for comparison. The Model III (M3) is based on the relevance vector machine to train the capacity model. The experimental results are shown in Figs. 7,  8 and 9, the statistical results are listed in Tables 3, 4,  5.

For the B5 battery, the predicted capacity of proposed M1 is closer to the actual capacity with a narrower lower CI compared with other methods. According to Table 3, the RMSEs of M1 at different *T* are around 0.02, which is closer to 0. The results indicate that the proposed M1 could be less affected by starting prediction points as well as more stable prediction performance. The AEs of M1 are less than 4, the RAs are higher than 0.9, and the CIs are less than 5, which proves the effectiveness of the proposed method. For the upper bound of CIs, which is calculated from the regeneration points (Reg-Points) of capacity, fluctuates continuously to assess the impact of the capacity regeneration. As can be seen from Fig. 7 to Fig. 9, when there is a significant capacity regeneration in the actual capacity, the upper bound almost coincides with it. When the capacity regeneration is less, the trend of upper bound is still similar to the trend of the regenerated capacity. It should be noted that when $T=70$, the CIs of RUL by the proposed method M1 do not include the final predicted RUL, precisely because the upper bound of the CI accurately captures the regenerated capacity, and the result can serve as an early warning. Therefore, the upper bound can provide a guidance to improve the efficiency of battery utilization.

**Table 4:** Experimental results of RUL online prediction for B6 battery.

*T*	Method	$\text {RUL}_{\text {true}}$	$\text {RUL}_{\text {pre}}$	RMSE	AE	RA	Uncertainty	CI
70	M1	39	34	0.0398	5	0.8718	[28 41]	13
	M2		37	0.0421	2	0.9487	[28 50]	22
	M3		21	0.1024	18	0.5385	[12 30]	18
80	M1	29	27	0.0338	2	0.9310	[22 25]	3
	M2		27	0.0403	2	0.9310	[21 36]	15
	M3		18	0.0609	11	0.6207	[6 32]	26
90	M1	19	17	0.0380	2	0.8947	[11 20]	9
	M2		15	0.0336	4	0.7895	[10 23]	13
	M3		12	0.0441	7	0.6316	[4 28]	24

For the B6 battery, according to Table 4, the proposed method M1 has a narrower CI than the comparison methods. And for the proposed M1, the RMSEs are less than 0.04, the AEs are less than 5. When $T=70$, although the AE of M1 is higher than that of M2 by 3, the significantly narrower CI of M1 includes the actual regenerated capacity. When $T=90$, the RMSE of the proposed method is 0.004 higher compared to M2, but its AE is smaller and the upper bound of CI almost captures the capacity regeneration at the starting point of the prediction, which is beneficial for the battery management system to make timely decisions before battery failure. Although the proposed method can accurately predict RUL and deal the effect of capacity regeneration, the predicted capacity at the end of the cycle deviates from the actual capacity, due to the observation of UKF as an output of $\text {Model}_{\text {GPR}}$, which constrains the accuracy of the predicted capacity as well as the CIs.

According to the prediction results of B7 battery from Fig. 7 to Fig. 9, the capacity degradation curves are accurately obtained by the proposed M1, and the lower prediction CI is narrower. Table 5 shows the comparative results for the B7 battery (Note: ’/’ indicates that the capacity predicted by the method did not reach the failure threshold of capacity). As shown in Table 5, compared with M2 and M3, the proposed M1 has the RMSEs lower than 0.0223 at different *T* with more stable prediction performance. The AEs of the proposed M1 are no more than 3, and the RAs are higher than 0.91 with a confidence interval of less than 13. The upper bound of CI obtained by the proposed method M1 can effectively reflect the impact of capacity regeneration, especially when $T=90$ and the capacity regeneration occurs at the starting prediction point. The experimental results prove the advantages of the proposed method.

**Table 5:** Experimental results of RUL online prediction for B7 battery.

*T*	Method	$\text {RUL}_{\text {true}}$	$\text {RUL}_{\text {pre}}$	RMSE	AE	RA	Uncertainty	CI
70	M1	56	58	0.0223	2	0.9643	[49 62]	13
	M2		36	0.0788	20	0.6429	[29 47]	18
	M3		62	0.0576	6	0.8929	[43 86]	43
80	M1	46	45	0.0161	1	0.9783	[37 49]	12
	M2		32	0.0441	14	0.6957	[25 43]	18
	M3		60	0.0647	14	0.6957	/	/
90	M1	36	33	0.0136	3	0.9167	[27 33]	6
	M2		28	0.0277	8	0.7778	[21 36]	15
	M3		34	0.0450	2	0.9444	[17 53]	36

**Figure 10:**
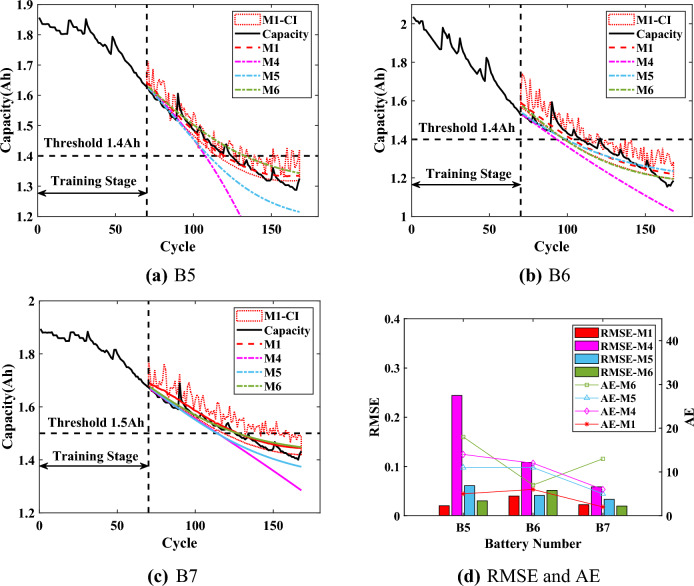
Prediction results of RUL when $T=70$.

**Figure 11:**
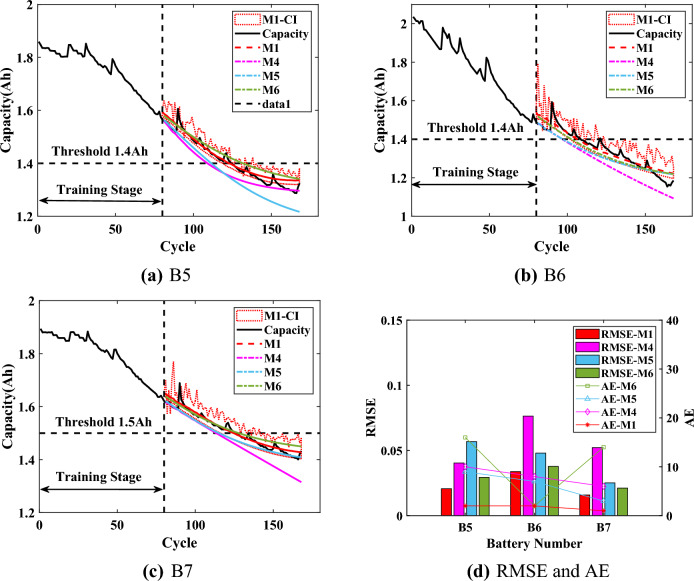
Prediction results of RUL when $T=80$.

**Table 6:** Experimental results for B5 battery.

*T*	Method	$\text {RUL}_{\text {true}}$	$\text {RUL}_{\text {pre}}$	RMSE	AE	RA
70	M1	55	51	0.0203	4	0.9273
	M4		41	0.2442	14	0.7455
	M5		44	0.0611	11	0.8000
	M6		73	0.0302	18	0.6727
80	M1	45	43	0.0208	2	0.9556
	M4		35	0.0404	10	0.7778
	M5		36	0.0568	9	0.8000
	M6		61	0.0294	16	0.6444
90	M1	35	34	0.0193	1	0.9714
	M4		60	0.0510	25	0.2857
	M5		48	0.0241	13	0.6286
	M6		46	0.0218	11	0.6857

**Figure 12:**
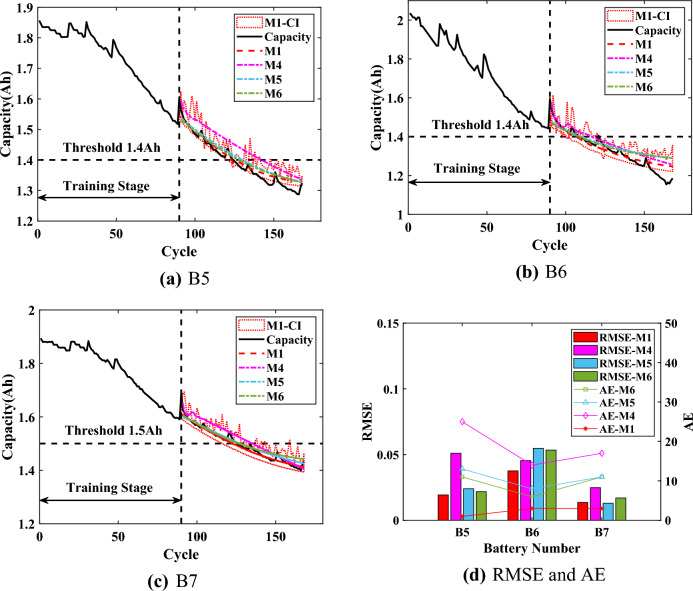
Prediction results of RUL when $T=90$.

Considering the application of the proposed method M1 to online RUL prediction, the experimental time of M1 and M2 was used to evaluate the computational burden. The methods were experimented on a personal computer with an AMD R7-5800H processor and 16 GB RAM. The prediction time of B6 ($T=70$) was taken as an example. And the average time for model training was 3.515s with a variance of 7.011E-03 for the 15 experiments performed by the comparison method M2. The proposed M1 needed to perform SHAP analysis on top of training as well as capacity regeneration capture with an average time of 1.257s and a variance of 4.735E-03. Although the method M1 needed to add the approximately 36% experimental time, its prediction results (RMSE and AE) were significantly better than those of the comparison methods. And the computational burden of the proposed method M1 was acceptable compared to the time period of 13000-14000s for a single cycle of the battery. To further verify the proposed method M1, the method M1 is compared with the mainstream online methods of step prediction, including the GPR (M4)^45^, the bidirectional long short-term memory networks (M5)^21^, and the method for the combination of empirical mode decomposition, long short-term memory and GPR (M6)^46^. The results are shown in Figs. 10,  11 and  12, and the statistical results are listed from Table 6 to Table  8.

When $T=70$, the performance of M1, M5 and M6 is stable. According to Fig. 10, the results of the proposed method M1 are significantly better on the two batteries B5 and B7. The RMSEs are from 2% to 4%, which illustrates that the overall prediction performance of M1 is better, and the predicted capacity is closer to the actual capacity. Meanwhile, the AEs of the three batteries are 4, 5 and 2. The AEs, while corroborating the prediction accuracy of capacity, prove that the predicted RUL of the proposed method M1 is reliable. As shown in Fig. 10b, the results of M5 are covered by the prediction confidence intervals of M1. But the upper CI of the proposed M1 is closer to the actual regenerated capacity and the predicted capacity is more accurate, which can ensure the reliable implementation of the threshold $Ca{{p}_{EOL}}$.

When $T=80$, all RMSEs are less than 8%, and the predicted capacity of all methods is more accurate compared to the results of $T=70$. As shown in Fig. 11d, the RMSEs and AEs of the proposed method M1 are significantly smaller than those of other methods especially for the B5 battery and the B7 battery. For the B5 battery, the AE of proposed M1 is only 2, while the AEs of the other methods are at least 9, which is significantly higher than the AE of M1. For the B7 battery, the proposed method M1 can accurately predict the battery capacity with the RMSE of 1.61% and the corresponding AE is only 1, which indicates that the predicted capacity almost coincides with the actual capacity. The results illustrate that the proposed method M1 can accurately predict RUL with strong stability.

**Table 7:** Experimental results for B6 battery.

*T*	Method	$\text {RUL}_{\text {true}}$	$\text {RUL}_{\text {pre}}$	RMSE	AE	RA
70	M1	39	34	0.0398	5	0.8718
	M4		27	0.1084	12	0.6923
	M5		34	0.0412	5	0.8718
	M6		32	0.0514	7	0.8205
80	M1	29	27	0.0338	2	0.9310
	M4		21	0.0764	8	0.7241
	M5		22	0.0480	7	0.7586
	M6		27	0.0378	2	0.9310
90	M1	19	17	0.0380	2	0.8947
	M4		33	0.0454	14	0.2631
	M5		27	0.0548	8	0.5789
	M6		25	0.0534	6	0.6842

When $T=90$, the AEs of the proposed M1 are 1, 2 and 3 for the three batteries, which is significantly better than the comparison methods. From Fig. 12, it can be seen that when the predicted capacity of the comparison methods is closer to the actual capacity, the corresponding curves all overlap with the confidence intervals of M1 to a large extent. These results firstly illustrate the accuracy of proposed method M1. And due to the different learning abilities of different methods for the overall degradation trend of capacity and local regeneration phenomenon, the results also prove the reliability of the proposed method M1 for RUL prediction. In addition, the proposed method M1 can accurately obtain and handle the capacity regeneration that occurs at the starting prediction point for all three batteries, with the upper bound of CIs that almost coincides with the regenerated capacity. The results prove the effectiveness and advantage of the proposed interpretable online prediction method for RUL.

**Table 8:** Experimental results for B7 battery.

*T*	Method	$\text {RUL}_{\text {true}}$	$\text {RUL}_{\text {pre}}$	RMSE	AE	RA
70	M1	56	58	0.0223	2	0.9643
	M4		50	0.0586	6	0.8929
	M5		51	0.0332	5	0.9107
	M6		69	0.0196	13	0.7679
80	M1	46	45	0.0161	1	0.9783
	M4		40	0.0522	6	0.8696
	M5		43	0.0252	3	0.9348
	M6		60	0.0212	14	0.6957
90	M1	36	33	0.0136	3	0.9167
	M4		53	0.0248	17	0.5278
	M5		47	0.0130	11	0.6944
	M6		47	0.0170	11	0.6944

## Conclusion

In this paper, an interpretable online prediction method for RUL of lithium-ion batteries has been proposed. The proposed method firstly extracts four appropriate health factors to comprehensively characterize the battery aging, preparing for online prediction. Secondly, the proposed method has been used to construct a hybrid framework of SVR, GPR, UKF and SHAP explainer to achieve the interpretable and accurate online prediction of RUL. Moreover, it obtains the narrower lower prediction CI and the upper bound of CI which reliably reflects the capacity regeneration. Finally, the verification experiments are performed using NASA data sets. The RMSEs of prediction results are less than 4%, and the maximum AE is 5. The experimental results illustrate that the redundant information of training data can be removed by the proposed method, and the method will add the information of random capacity regeneration. Thus, the constructed HIs and the capacity model can effectively retain the capacity information with the confidence intervals reflecting the regenerated capacity. The suggested method has the potential to facilitate timely maintenance of lithium-ion batteries and enhance battery utilization efficiency.

The suggested method has demonstrated great prediction capability. However, it still requires sufficient amount of high-quality training data with reliable processing hardware. The future research of this work is to reduce the data dependency of the method and increase the robustness to variable operation conditions by transfer learning. High-rate charging and low-temperature are highly investigated operation conditions, which lead to nonlinear variations in battery characteristics that have been difficult to predict in RUL. Therefore, the subsequent study will specifically focus on these two extreme conditions. In addition, the batteries are usually combined into battery packs by series-parallel connections in devices. Therefore, research on battery packs also needs to be considered in the prediction.

## Data Availability

Te datasets used and/or analyzed during the current study are available from the corresponding author upon reasonable request.
